# Diagnosis and Treatment of Nonfunctioning Pituitary Adenomas with Thyroid Disorders

**DOI:** 10.1155/2023/2846601

**Published:** 2023-03-27

**Authors:** Xu Wang, Mingchu Li, Xiaohai Liu, Jiantao Liang, Hongchuan Guo, Ge Chen

**Affiliations:** ^1^Department of Neurosurgery, Xuanwu Hospital, Capital Medical University, Beijing, China; ^2^International Neuroscience Institute (China-INI), Beijing, China

## Abstract

**Objective:**

Patients with nonfunctioning pituitary adenoma (NFPA) can present with different types of thyroid disorders, which are easily misdiagnosed or missed and can even result in serious clinical consequences. This study was to summarize the different types of thyroid disorders in patients with NFPA with the aim of providing references for the diagnosis and treatment of such patients.

**Materials and Methods:**

The data of pituitary adenoma (PA) patients who underwent surgical treatment at Xuanwu Hospital, Capital Medical University, from 2017 to 2021 were retrospectively analyzed, and NFPA patients with preoperative thyroid disorders were screened out to analyze their imaging, endocrine, treatment, and prognosis data. Also, thyroid disorders were classified to summarize diagnostic methods and treatment principles for different types of thyroid disorders.

**Results:**

A total of 399 NFPA patients were included in this study, of which 67 (16.8%) had thyroid disorders before surgery. Fifty-four patients had (13.5%) central hypothyroidism (CH) caused by NFPA and were treated with levothyroxine (L-T4) supplementation before and after operation. Eleven patients (2.8%) had primary hypothyroidism and were treated with L-T4 during the perioperative period, and long-term treatment of primary hypothyroidism was provided after surgery. Two NFPA patients (0.5%) were combined with primary hyperthyroidism and treated with medication for primary hyperthyroidism after tumor resection.

**Conclusion:**

Thyroid disorders are relatively common in patients with NFPA, but are difficult to be diagnosed due to their different types. CH is the most common type of thyroid disorder, which requires aggressive L-T4 supplementation during the preoperative period. The primary disease of the thyroid gland is easily missed when NFPA is combined with primary hypothyroidism or primary hyperthyroidism, and the thyroid function test results require to be analyzed carefully for continued treatment for thyroid disease after resection of the NFPA.

## 1. Background

Normal thyroid function is essential for maintaining normal metabolism and growth and development of the body. The normal function of the hypothalamic-pituitary-thyroid axis is susceptible to a variety of factors. Patients with pituitary adenoma (PA) may suffer from thyroid disorder due to a variety of factors, which results in difficulties in diagnosis and possible delay in treatment if misdiagnosed. PA can cause compression of the normal pituitary gland or its blood supply arteries, which can impair the secretory function of thyrotropin (TSH) cells and thus result in central hypothyroidism (CH) [1–3]. A variety of hypothalamic and pituitary disorders can cause CH, with PA being the most common cause. The prevalence of CH is low in the general population, approximately 1 : 20,000–1 : 120,000 [3]. However, CH is common in patients with PA. According to Tetsuya Takamizawa et al. the prevalence of CH in patients with nonfunctioning pituitary adenoma (NFPA) can be up to 24% [4].

NFPA are common intracranial tumors, and primary hypothyroidism is also a common condition with the prevalence of 1%-2% in the population [5]. Theoretically, NFPA with primary hypothyroidism is frequently observed. NFPA with primary hypothyroidism is prone to misdiagnosis as CH, but the two needs to be treated according to different principles [6]. Similarly, primary hyperthyroidism is a common condition with the prevalence of approximately 0.5% in the population [7]. NFPA with primary hyperthyroidism may be misdiagnosed as TSH-secreting pituitary adenoma (TSHoma), thus resulting in delayed treatment of primary hyperthyroidism. Despite that NFPA with primary hypothyroidism and primary hyperthyroidism are rarely reported, it is indeed frequently observed in clinical practice. The presence of NFPA is likely to mask a primary disease of the thyroid gland and it is necessary to diagnose it early and promptly to enable correct treatment. In conclusion, patients with NFPA present with a wide variety of thyroid disorders, resulting in difficulty in diagnosis. For these reasons, data from patients with NFPA admitted to our center in the past 5 years were retrospectively analyzed and different types of thyroid dysfunction were analyzed so as to summarize corresponding diagnosis and treatment options.

## 2. Methods

Data of patients with PA who underwent surgery at Xuanwu Hospital from January 2017 to December 2021 were retrospectively analyzed, including clinical data, endocrine data and imaging data, and pathological findings. The inclusion criteria included pituitary lesions on brain MRI and NPFA confirmed by postoperative pathology, thyroid function tests performed before and after surgery with complete data and signed informed consent. Exclusion criteria included previous hypothalamic, pituitary surgery, or radiation therapy; previous thyroid surgery or radiation therapy; treatment with L-T4 for more than 1 week within 2 months before admission; combined hematologic disease, combined other malignancies, and combined other serious immune system diseases. From the above patient data, patients with preoperative thyroid disorders were selected, and their imaging, endocrine, treatment, and pathological data were analyzed, and thyroid disorders were classified to summarize the diagnostic methods and treatment principles for different types of thyroid disorders.

### 2.1. Statistical Analysis

Continuous variables were presented as the mean ± standard deviation, and categorical variables were presented as percentages. Baseline characteristics of patients were analyzed and compared between the two groups using descriptive statistics. For qualitative variables, data were compared using the *χ*2 and Fisher's exact tests when appropriate; for quantitative variables, data were compared using the two-sided unpaired Student *t* and the Mann–Whitney *U* tests when appropriate. Normality of quantitative variables was determined by the Shapiro–Wilk test. All calculations were performed using the IBM SPSS software, version 28.0 (IBM Corporation, Armonk, NY, USA). A value of *p* < 0.05 was considered statistically significant.

## 3. Results

From January 2017 to December 2021, 665 patients underwent PA resection in our center, 266 patients were excluded according to the inclusion preoperative criteria, and a total of 399 patients were included in this study. Of the 399 patients with NFPA enrolled, a total of 67 patients (16.8%) had preoperative thyroid disorders. Of the 67 patients with thyroid disorders, CH occurred in 54 patients (80.6%), NFPA with primary hyperthyroidism in 2 (3.0%) patients, and NFPA with primary hypothyroidism in 11 patients (16.4%).

### 3.1. Central Hypothyroidism

**Figure 1:**
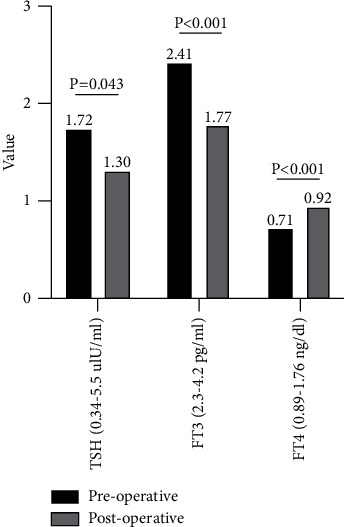
The preoperative and postoperative thyroid function results of the 54 NFPA patients combined with CH.

CH is diagnosed based on a decrease in free thyroxine (FT4) and normal or decreased thyrotrophin (TSH) [3]. Patients who were diagnosed with CH upon admission were treated preoperatively with L-T4 based on the patient's weight and FT4 results. The prevalence of CH was 13.5% in the 399 enrolled patients. The maximum tumor diameter was measured on the preoperative brain MRI. There were 179 patients with tumor diameter larger than 20 mm and 220 patients with tumor diameter less than 20 mm. The mean tumor diameter of 54 patients with CH was significantly larger than the 345 patients without CH (23.3 mm vs. 20.9 mm, *p* = 0.021). In the group of tumor diameter larger than 20 mm, the prevalence of CH was significantly higher than the group of tumor diameter less than 20 mm (18.4% vs. 9.5%, *p* = 0.01). The preoperative clinical data, thyroid function tests, including TSH, free thyronine (FT3), and FT4 of all 54 patients with CH are illustrated in Table 1. Among the 54 patients, 53 were treated with endoscopic surgery and 1 with craniotomy to maximize resection of the tumor on the basis of protecting the internal carotid artery, optic nerve, and pituitary tissue. TSH and FT3 decreased significantly after surgery compared with those before surgery, while FT4 increased significantly compared with those before surgery (Figure 1). After surgery, 2 patients had intracranial infection and 1 had epistaxis, all of which recovered smoothly, and 1 had cerebral infarction. After surgery, all patients continued L-T4 supplementation and had their thyroid function rechecked after 1 month and the dose of L-T4 adjusted as needed.

### 3.2. NFPA Combined with Primary Hypothyroidism

**Figure 2:**
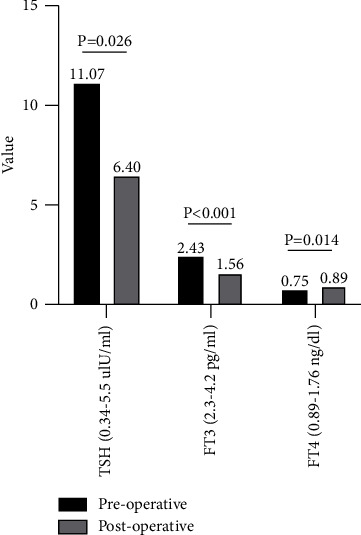
The preoperative and postoperative thyroid function results of the 11 NFPA patients combined with primary hypothyroidism.

The diagnostic criteria were pathologically confirmed NFPA, elevated TSH and decreased FT4 with or without decreased FT3 indicated by endocrine laboratory tests. In this group, 11 patients had combined primary hypothyroidism. The clinical and thyroid function data are summarized in Table 2. All 11 patients were diagnosed with primary hypothyroidism during hospitalization. The proportion of patients with primary hypothyroidism was 2.8% in this group of patients with NFPA. All 11 patients underwent endoscopic surgery and they had a significant decrease in TSH and FT3 and a significant increase in FT4 after surgery compared to those before surgery (Figure 2). All 11 patients presented with no postoperative complications, and long-term oral L-T4 was continued after surgery for primary hypothyroidism.

### 3.3. NFPA with Primary Hyperthyroidism

The diagnostic criteria were pathologically confirmed NFPA, decreased TSH and elevated FT4 and FT3 indicated by endocrine laboratory tests, and exclusion of the effect of oral administration of L-T4. In this group, two patients were combined with primary hyperthyroidism, with a prevalence of 0.5%. Two patients mainly suffered from visual decrease caused by oppressing of optic nerve by tumor, but slight hyperhidrosis and palpitation were not paid attention to. After admission, the patients were diagnosed with primary hyperthyroidism based endocrine tests, and underwent surgeries under trans-nasal endoscope. One patient developed cerebrospinal fluid leakage after surgery and was cured after lumbar drainage for 1 week. Postoperatively, treatment of primary hyperthyroidism was continued and thyroid function was monitored. The clinical data and thyroid function results of the 2 patients before and 3–5 days after surgery are described in Table 3.

The diagnosis and treatment principles of different types of thyroid disorders in combination with NFPA are shown in Table 4.

## 4. Discussion

Patients with PA often present with thyroid disorders and have various manifestations, resulting in some confusion in diagnosis and treatment. Patients with functional PA are prone to central hypothyroidism, or varying degrees of FT3 and FT4 changes, due to the complex effects of growth hormone, cortisol, and prolactin on the hypothalamic-pituitary-thyroid axis [8–11]. Patients with TSH adenoma may experience significant hyperthyroidism, with significantly elevated FT4 and TSH, and secondary-thyroid lesions. Thyroid disease is more common in patients with growth hormone adenomas, which is often difficult to be interpreted with thyroid function test results [12–15]. Moreover, endocrine cells in the pituitary gland may be damaged during surgery, and hypothyroidism may also occur after surgery. In this study, thyroid function in patients with NFPA not disturbed by surgery or drugs was analyzed to evaluate the type and incidence of possible thyroid function abnormalities so as to accumulate experience for treatment.

### 4.1. Central Hypothyroidism

CH is common in patients with PA and is easily diagnosed. Mukai et al. noted that CH is related to the volume of the tumor, and a large PA is more likely to cause CH [16]. This is similar to our results. We found that the prevalence of CH is significantly higher in the patients with larger tumor diameter. On the other hand, patients with adrenocorticotropic hormone secreting PA are prone to CH due to a decrease in TSH and FT4 resulting from the decreased responsiveness of TSH to thyrotropin-releasing hormone and the inhibitory effect of increased cortisol on the hypothalamic-pituitary-thyroid axis [8–10]. The incidence of CH in our group was 13.5%, slightly lower than that reported in previous studies. Regardless of the type of PA patients, the occurrence of CH should be paid attention to, and adequate L-T4 supplementation is required before surgery to ensure perioperative safety. Thyroid function should be closely monitored after surgery, and L-T4 supplementation should be continued until the function of the hypothalamic-pituitary-thyroid axis is completely recovered.

In this group of patients, 54 patients with CH had insignificant decrease in TSH after surgery compared with that before surgery (Figure 1), probably due to the damage to the adenohypophysis during the intraoperative resection of the tumor, and a small amount of TSH cells were destroyed. FT4 was slightly elevated after surgery compared to that before surgery, which may be due to two factors. First, it is considered that FT4 was slightly elevated due to L-T4 supplementation in perioperative period. Furthermore, it might also be due to recovery of hypothalamic-pituitary-thyroid axis function after surgery.

### 4.2. NFPA with Primary Hypothyroidism

The possibility of combined primary hypothyroidism should be considered when patients with PA present with elevated TSH and decreased FT4, and such patients may also be misdiagnosed as patients with TSH adenomas. Primary hypothyroidism could be easily missed in patients with NFPA. Primary hypothyroidism is also common in the population, with a prevalence of more than 1% [17, 18]. In this study, the prevalence of NFPA with primary hypothyroidism was 2.8%, which is higher than that in the general population. However, NFPA with primary hypothyroidism is rarely reported. Primary hypothyroidism may cause pituitary hyperplasia and even secondary PA through a negative feedback mechanism, and PA also often causes thyroid disorders [19]. As a result, it is difficult to clarify the correlation between thyroid diseases and PA in these patients. In a word, NFPA with primary hypothyroidism is frequently observed in clinical practice and must be clearly diagnosed and cannot be misdiagnosed so as to avoid long-term hypothyroidism due to delayed treatment, thus affecting the patient's quality of life.

### 4.3. NFPA with Primary Hyperthyroidism

NFPA with primary hyperthyroidism needs to be distinguished from the following two conditions: One is CH, which is also characterized by decreased TSH and is more common in clinical practice, but is presented with decreased FT4, so it is easy to be differentiated. The other is TSHoma, which is characterized by elevated TSH and FT4, and TSHoma is very rare, accounting for only 0.5%–3% of all PA [20, 21], and patients mostly present with significant hyperthyroid symptoms, which is favorable for preoperative differential diagnosis and can also be confirmed by postoperative pathological examination. NFPA with primary hyperthyroidism is rarely studied. It should not be ignored that both NFPA and primary hyperthyroidism are relatively common disorders and it is entirely possible for them to be combined. The prevalence of combined primary hyperthyroidism in patients with NFPA was 0.5% in this group of patients, compared with 0.5%–1.0% in the general population [22–24].

If just the symptoms are concerned before surgery and abnormal thyroid function is not detected, it is likely to lead to missed diagnosis of primary hyperthyroidism and thus delay the treatment. When NFPA are associated with primary hyperthyroidism, medication for hyperthyroidism should be used to control thyroid function within a safe range before surgery. Multidisciplinary treatment is helpful to develop the best treatment regimen.

## 5. Conclusion

Thyroid disorders are relatively common in patients with NFPA, but are difficult to be diagnosed due to their different types. CH is the most common type of thyroid disorder, which requires aggressive L-T4 supplementation during the preoperative period. The primary disease of the thyroid gland is easily missed when NFPA is combined with primary hypothyroidism or primary hyperthyroidism, and the thyroid function test results require to be analyzed carefully for continued treatment for thyroid disease after resection of the NFPA.

## Figures and Tables

**Table 1:** The clinical information and preoperative thyroid function results of the 54 NFPA patients combined with CH.

Characteristics	Number
Age	51.9 ± 12.4
M/F	37/17
Duration of symptoms (months)	9.6 ± 7.4
TSH (0.34–5.5 *μ*IU/ml)	1.72 ± 0.95
FT3 (2.3–4.2 pg/ml)	2.41 ± 0.45
FT4 (0.89–1.76 ng/dl)	0.71 ± 0.10

**Table 2:** The clinical information and preoperative and postoperative thyroid function results of the 11 patients combined with primary hypothyroidism.

Characteristics	Number
Age	53.8 ± 10.1
M/F	6/5
Duration of symptoms (months)	32.0 ± 15.3
TSH (0.34–5.5 *μ*IU/ml)	11.07 ± 6.78 (5.69–24.18)
FT3 (2.3–4.2 pg/ml)	2.43 ± 0.47 (1.90–3.38)
FT4 (0.89–1.76 ng/dl)	0.75 ± 0.18 (0.36–0.84)

**Table 3:** The clinical information and preoperative thyroid function results of the two patients combined with primary hyperthyroidism.

Characteristics	Number
Age	52.5
M/F	2/0
Duration of symptoms (months)	2.0
Preoperative	
TSH (0.34–5.5 *μ*IU/ml)	0.004
FT3 (2.3–4.2 pg/ml)	10.90
FT4 (0.89–1.76 ng/dl)	2.76
Postoperative	
TSH (0.34–5.5 *μ*IU/ml)	0.005
FT3 (2.3–4.2 pg/ml)	6.85
FT4 (0.89–1.76 ng/dl)	3.56

**Table 4:** The diagnosis and therapy strategies of different types of thyroid dysfunction that could be combined in patients with NFPA.

Type of thyroid dysfunction	TSH	FT3	FT4	Danger	Therapy
CH	Normal or decrease	Normal or decrease	Decrease	Long-term hypothyroidism	Surgery + L-T4
NFPA combined with primary hyperthyroidism	Decrease	Elevate	Elevate or normal	Ignorance of hyperthyroidism	Surgery + long-term treatment of hyperthyroidism
NFPA combined with primary hypothyroidism	Elevate	Normal or decrease	Decrease	Misdiagnosed as TSHoma; ignorance of hypothyroidism	Surgery + long-term L-T4

## Data Availability

The data are provided in the Supplementary Information files.
